# Construction of Relationship Model between College Students' Psychological Status and Epidemic Situation Based on BP Neural Network

**DOI:** 10.1155/2022/5115432

**Published:** 2022-02-22

**Authors:** Shuguang Yao

**Affiliations:** Anyang Normal University, Anyang 455000, China

## Abstract

In view of the impact of COVID-19 on the mental health of college students, this paper proposes a study on the relationship between psychological status and epidemic situation of university students based on BP neural network, so as to provide theoretical basis for universities to take targeted mental health education. This paper investigates the effects of COVID-19 on the psychological emotions of college students. According to the behavior and psychological characteristics of college students, the relevant investigation results are obtained through event monitoring, early warning, and usual performance, and a relationship model between college students' psychological status and epidemic situation based on BP neural network is constructed. This paper studies several factors through the relationship model and uses the principal component analysis method to analyze the impact of various factors on college students' psychology. According to the model prediction and result analysis, it concluded that the influence of COVID-19 should focus on improving the professional quality, physical quality, humanistic quality, and moral quality of university students, so as to improve the stability of colleges and universities in the event of public health emergencies. The model constructed in this paper can provide reference for carrying out mental health education and formulating effective intervention programs.

## 1. Introduction

The COVID-19 epidemic is a global public health emergency, and some people will have a strong stress response and show obvious symptoms of anxiety and depression. Facing the COVID-19 epidemic, the group of college students will have a stress response, which will have a negative impact on their normal life and study, such as a decrease in outing activities, a decrease in communication, and a change in learning methods. Therefore, college students are prone to different levels of emotional problems, and the duration is longer.

In order to win the battle against the epidemic and respond to the call of the country, everyone has been quarantined at home. The development of the epidemic, the restriction of activities, and the changes in the original life state have made everyone anxious and fearful. For college students who are unable to go to school normally, the continuous expansion of the epidemic has led to uncertain return to school time, home study has led to changes in learning styles, and the employment and graduation issues that graduating students worry about have increased the anxiety and depression of college students, which in turn affects their mental health. Therefore, various universities and the education department have issued guidance manuals on the mental health of college students during COVID-19 to carry out psychological crisis interventions in public health emergencies.

Some scholars have also conducted research on the psychological problems and protection of college students in major epidemics and published relevant results. The results are mainly concentrated in the following three aspects. The first is the investigation of the psychological status of college students under the background of the new crown epidemic [1]. Many investigations and studies believe that the new crown epidemic has a significant impact on the psychology of college students, causing psychological problems such as panic, anxiety, anger, and stress among college students. Therefore, schools and society should strengthen the cultivation of college students' psychological quality in response to public health emergencies and improve their psychological endurance [2]. The second is the relationship between the awareness of the new crown epidemic and the physical and mental health of college students. The results of data analysis show that the cognitive level of COVID-19 is significantly related to the physical and mental health of college students. The education department should strengthen targeted publicity and education and strive to improve college students' cognitive level of the epidemic and enhance their physical and mental health [3].

Some scholars found in the investigation that the anxiety level of college students was higher than the norm during the epidemic prevention and control period. By summarizing the existing research results, it is found that its characteristics mainly have two scores [4]. One is that some of the results only focus on quantitative analysis of the psychological problems of college students under the background of major epidemics but lack qualitative research on the specific manifestations, characteristics, and effects of psychological problems. Second, some scholars only pay attention to presenting the psychological problems of college students in their research and make suggestions, but there are not many specific measures for the psychological protection of college students under the background of major epidemics.

The report of the 19th National Congress of the Communist Party of China clearly stated that a healthy China strategy should be implemented, and the policy of “prevention first, combined prevention and treatment” was advocated. The epidemic has caused people to experience anxiety, depression, and other emotions, and students' psychological and behavioral problems have also begun to become prominent. How to motivate college students to get rid of the trouble of bad emotions and integrate into college life as soon as possible, and to pose new challenges to college students' physical health education work, has also become an important topic for building a healthy Chinese background. First of all, doing a good job of mental health education for college students in the context of epidemic prevention and control is an important content and internal need for the development of the discipline of mental health education, and it is also an important work to effectively improve the overall level of psychological services. The second is the core task of ideological and political education in colleges and universities in response to the epidemic, which is of great significance to maintaining the stability of the campus and creating a civilized campus [5].

Contemporary college students are in a special period of physical, psychological development and maturity and are in a special stage of life development and have a special social environment and social norms. In this special group, there are many special stressors specifically for them. The stress of foreign college students mainly comes from study [6], social and emotional state or environment [7], personal aspects [8], including examination [9], competition, time, teachers, classroom environment [10], employment, parental relationships, close relationships, economic problems [11], interpersonal relationships, living conditions, appearance, etc. Domestically, it comes from learning troubles, personal worries, and negative life events [12], including study, employment, interpersonal relationships, life, romantic relationships [13], economy, society, family [14], examination, life, learning environment [15], future, ability, personal health [16], competition, etc. The pressure sources of college students at home and abroad are similar. The emergence of these problems has formed the unique characteristics of psychological activities of this group. The results of psychological surveys of college students show that many colleges are living with psychosomatic disorders, and stress is one of the important factors that affect their health [16]. Therefore, understanding the basic conditions and influencing factors of college students' behavior and psychological stress is the basis for further research on college students' behavior and mental health at present and in the future and is the key to formulating plans and implementation steps for college students' physical and mental development. Based on this far-reaching significance, it focuses on systematically elaborating the factors affecting college students' behavior and psychological stress [17].

College students are in an important stage of rapid physical and mental development and change, complex and changeable emotional response, self-consciousness from semi-independence to independence, and social role transition. They lack of understanding of public health emergencies, which is easy to affect their psychological state. It is known from the existing research that the research on the relationship between college students' psychological status and epidemic situation is not deep enough. Therefore, this paper proposes the construction of the relationship model between college students' psychological status and epidemic situation based on BP neural network.

## 2. BP Neural Network

Artificial neural networks (ANNs) are an algorithmic mathematical model that imitates the behavior characteristics of animal neural networks for distributed parallel information processing. At present, there are dozens of artificial neural network models with more applications, including BP neural network, Hopfield network, art network, and Kohonen network.

### 2.1. Neuron Model

**Figure 1:**
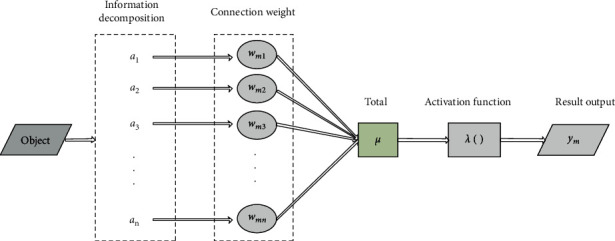
Structure diagram of neuron model.

BP neural network feeds back the error between the actual output value and the expected output value and then constantly trains and adjusts the model to reduce the error and make the prediction result of the model approach the actual situation. The basic component of BP neural network is neurons, which are connected with each other and transmit information through weighted connection channels. Neurons combine multiple received signals and then calculate the output results through the activation function, as shown in Figure 1.

The calculation formula of neuron output is as follows:

(1) $$\begin{array}{c} y_{m}=\lambda \left(\sum_{i=1}^{n}w_{mi}a_{i}-\mu \right), \end{array}$$

where *y*_*m*_ shows the output value after processing, *w*_*mi*_ denotes the connection weight of each transmission signal, *a*_*k*_ represents the input signal of the upper layer, *λ* represents the activation function of neurons, and *μ* is the threshold of neurons.

### 2.2. Construction of Neural Network Model

**Figure 2:**
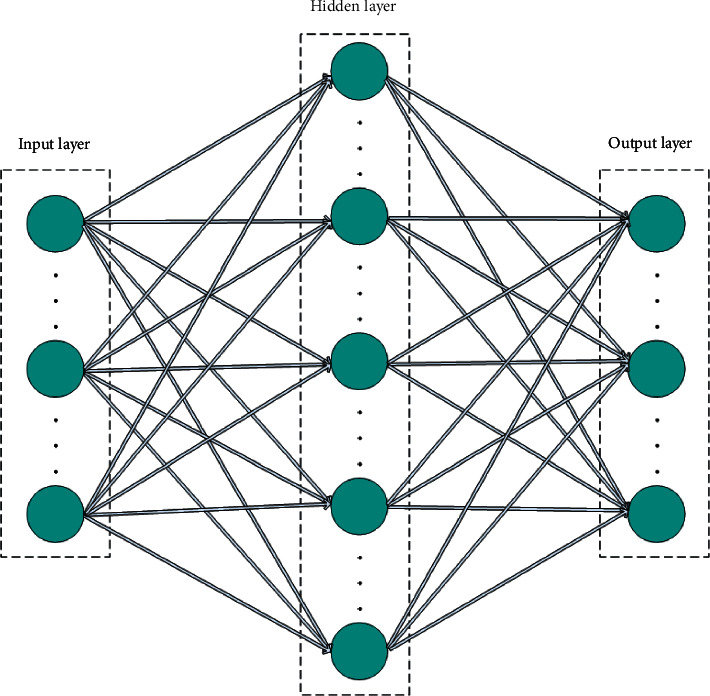
Structure diagram of neural network.

BP neural network is a three-layer structure (as shown in Figure 2), which is composed of input layer, output layer, and hidden layer. According to the different problems to be solved, the hidden layer of multilayered structure can be set, but the most classic is only one hidden layer, which makes it easy to understand the relationship between analog input and output. Each layer of BP neural network has multiple neurons, and their number is determined by a specific model, while the two neurons in the hierarchy are not interconnected. All neurons are connected with the nearby hierarchy through one-way connection, and the connected nodes interact through weight. When the signal enters the hidden layer through the input layer, the connection function will process the transmitted data, and then the hidden layer will transmit the processed data to the output layer of the neural network and finally get the output result.

### 2.3. Implementation Process of BP Neural Network

The input layer described in this paper includes 3 nodes: basic information of students, epidemic information, and psychological information.

The output layer has only label degree; that is, the output layer is one node. The number of nodes in the hidden layer is *l*, which is obtained through the analysis of training experiments.

In order to eliminate the influence of different dimensions in the three evaluation indexes of student number, epidemic severity, and psychological status, the data were standardized. The index variables are mapped to [0, 1] through normalization, and the formula is as follows:

(2) $$\begin{array}{c} q_{i}^{k}=\frac{Q_{i}^{k}-Q_{i\;\min}}{Q_{i\;\max}-Q_{i\;\min}}, \end{array}$$

where *q*_*i*_^*k*^(*k*=300) is the normalized data, *i*=1,2,3, *k*=1,2,…, 300, and *Q*_*i*max_ and *Q*_*i*min_ are the maximum and minimum values in the *k* -th original data. 80% of the data are randomly selected from the normalized data set as training data and the remaining 20% as test data.

Input the training data into the neural network, and the output value of the hidden layer can be obtained through equation (3), shown as follows:

(3) $$\begin{array}{c} h_{m}^{k}=\sum_{i=1}^{3}w_{im}^{\left(v\right)}Q_{i}^{k}+l_{m}^{\left(v\right)}, \end{array}$$

where *m*=1,2,…, *N*, *N* is the number of nodes in the hidden layer, *w*_*im*_^(*v*)^ is the connection weight between the input layer and the hidden layer, and *l*_*m*_^(*v*)^ is the threshold of the hidden layer.

In order to obtain better convergence effect and improve the accuracy of the model, Sigmoid function is introduced into the hidden layer, shown as follows:

(4) $$\begin{array}{c} p_{m}^{k}=\frac{1}{1+e^{-\sum_{i=1}^{6}w_{im}^{\left(v\right)}Q_{i}^{k}+l_{m}^{\left(v\right)}}}, \end{array}$$

where *p*_*m*_^*k*^ is the output value of the hidden layer and *k* is 900. Take the output value of the hidden layer as the input data of the output layer, and calculate the output value of the output layer by using equation (5), shown as follows:

(5) $$\begin{array}{c} Z^{k}=\sum_{i=1}^{3}w_{i}p_{i}^{k}+l_{i}. \end{array}$$

Among them, *w*_*i*_ is the connection weight, *l* is the output layer threshold, and *Z*^*k*^ is the output data of the output layer. In order to obtain better effect of the model, calculate the mean square error through equation (6), which is expressed as follows:

(6) $$\begin{array}{c} \text{Mean}=\frac{1}{900}\sum_{n=1}^{900}\left(Z^{n}-R^{n}\right). \end{array}$$

Among them, *R*^*n*^ is the actual value and *Z*^*n*^ is the predicted value. The gradient descent method is used to backpropagate the error, correct the connection weight and threshold of each layer, and stop training until the target accuracy or target training times are reached, which is shown as follows:

(7) $$\begin{array}{c} w_{N+1}=w_{N}-\theta \frac{\partial_{\text{Mean}}}{\partial_{w}},\\ S_{N+1}=S_{N}-\theta \frac{\partial_{\text{Mean}}}{\partial_{S}}, \end{array}$$

where *S*_*N*_ denotes the threshold of each layer, *w*_*N*_ represents the connection weight of each layer, *S*_*N*+1_ is the threshold correction value, *w*_*N*+1_ shows the correction value of connection weight, and *θ* is the learning rate.

## 3. Method and Modeling

### 3.1. Variables in Process

In order to discover and further explore the behavioral factors that affect college students under public health emergencies, this paper proposes corresponding solutions and countermeasures and uses more precise statistical analysis methods to study them. Path analysis is used to study the ways in which earlier variables affect subsequent variables, reveal the hierarchical relationship between factors, and suggest the existence of causal or related relationships. This paper screens out various factors affecting the peak behavior of the new crown epidemic at the 0.05 level. On this basis, this paper establishes the restriction mode of path analysis and obtains the path analysis diagram of the restriction mode to show the hierarchical logical relationship that affects the peak behavior of the new crown epidemic, to provide a basis for improving the behavior of the peak of the COVID-19 epidemic. In path analysis, the path coefficient is a standardized partial regression coefficient, the sign reflects the direction of action, and the absolute value reflects the degree of direct influence between levels. Secondly, the degree of indirect influence can be reflected by the size of the product of the corresponding path coefficients. The standard coefficient of overall influence is the sum of the product of the standard partial regression coefficients of each channel in the path, that is, the sum of direct and indirect influences [18].

In the questionnaire design, this study adds some inverse questions and similar questions. The purpose is to facilitate the screening of invalid questionnaires to prevent misjudgment of the measurement results. A total of 878 complete questionnaires are collected in this survey, and these 878 questionnaires are screened one by one, and invalid questionnaires are eliminated. The principle of eliminating questionnaires are as follows: one is to eliminate questionnaires with inconsistent answers before and after similar questions; the other is to eliminate questionnaires with the same answer for all questions, including inverse questions. This questionnaire is set to submit permissions, so there will be no missing questionnaires.

Based on existing mental health, psychology should actively take the responsibility of maintaining social mental health in response to new problems and new trends in society. The scale was compiled by Derogates and has been modified many times during its use. There are 90 self-assessment items in the SCL-90 scale. The nine factors tested are somatization, obsessive-compulsive symptoms, interpersonal sensitivity, depression, anxiety, hostility, horror, paranoia, and psychosis. In addition, it also includes 1 other factor, which mainly reflects sleep and diet. In general research, it is classified as the tenth factor. At the same time, this paper also designs 13 questions including demographic characteristics. This questionnaire uses a five-level score of 1 (never), 2 (very light), 3 (medium), 4 (heavier), and 5 (very heavy). Moreover, it takes the score of one or more factors in the 10 factors of the SCL-90 scale ≥3 as the standard for testing positive symptoms of mental health. Cronbach's *α* coefficient of the SCL-90 scale in this study was 0.922 [19].

The contents of the self-made questionnaire are as follows:

1. Basic information: school, grade, major, gender, age, ethnicity, whether it is an only child, physical condition, whether to serve as a student cadre, learning situation during school, relationship with classmates and teachers, hometown location, hometown new crown epidemic situation, etc.
2. Coping style: In the standard scale-coping style questionnaire, according to the tendency of individual coping behavior types, it is divided into mature and immature types. The problems reflecting the six coping factors of “withdrawal,” “fantasy,” “self-blame,” “seeking for help,” “rationalization,” and “solving problems” are selected separately: “Borrowing cigarettes or alcohol to dissipate sorrow”-“Retreat,” “I hope that I have solved the problem facing”-“Fantasy,” “Always blame oneself”-“self-blame,” “Buried unpleasant things in one's heart”-“seeking for help,” “calm down the troubles”-“rationalize,” “try to see the good side of things”-“solve problems.” Each has four options: “Never,” “Occasionally,” “Sometimes,” and “Always.” The corresponding scores are 1–4 scores, and the cumulative scores are 6–24 scores. The higher the score, the more mature the college students are in responding to public health emergencies.
3. Behavioral and psychological investigations during the peak period of the COVID-19 epidemic: (1) Behavior survey: including personal hygiene habits: hand washing conditions, according to frequency from low to high, it is recorded as 1–3 scores; spitting and throwing garbage anywhere, according to the frequency of occurrence from quotient to low, it is recorded as 1–4 scores; dining in small restaurants outside: according to the frequency of occurrence, it is recorded as 1–5 scores. Improve self-immunity: Whether to pay attention to the diet and nutrition, whether to work and rest on time, whether to exercise: According to the frequency of occurrence from low to high, they are all recorded as 1–5 scores. (2) Psychological investigation (focusing on the investigation of psychological stress): including the frequency of wearing masks, whether you have ever taken healthcare products, whether you have ever taken drugs to prevent the COVID-19 epidemic, whether you are worried about your family members being infected, and whether you feel suffocated in public, etc.
4. Psychological investigation in the middle and late stages of the COVID-19 epidemic: (1) Anxiety state: In the standard scale—self-rating anxiety scale, we select six aspects of “anxiety,” “unfortunate premonition,” “fatigue,” “can't sit still,” and “sleep disorder,” respectively. Each item has four options: “No or Rarely,” “Sometimes,” “Often,” and “Always,” which are recorded as 1, 2, 3, and 4 scores, and the total score is 5–20 scores. The higher the score, the higher the degree of depression. (2) Depressive state: In the standard scale—self-rated depression scale, three questions of “depressive mood,” “irritability,” and “emptiness” are selected, respectively. Each item has four options: “No or Rarely,” “Sometimes,” “Often,” and “Always,” which are recorded as 1, 2, 3, and 4 scores, and the total score is 5–20 scores. The higher the score, the higher the degree of depression.
5. Behavioral and psychological investigations in the late stage of the COVID-19 epidemic: (1) Behavioral investigation: including the maintenance of personal hygiene habits: whether to spit and throw trash, whether to gather at a small restaurant, and how often to wash hands every day; improve self-immunity: whether you still pay attention to diet and nutrition, whether you still work on time, whether you still exercise, etc. (2) Psychological survey (focusing on the measurement of subjective feeling): After the COVID-19 epidemic is under control, whether students feel relaxed psychologically is divided into five levels: “not relaxed,” “a little relaxed,” “some relaxed,” “relatively relaxed,” and “completely relaxed.”
6. Survey of perceptions and knowledge during the occurrence and development of the new crown epidemic: it mainly involves (1) the communication between the school and the students: whether the students are satisfied with the various prevention and control measures taken by the school during the new crown epidemic (hereinafter referred to as “Opinions to the School”). (2) Students' social support system: How to resolve the psychological tension caused by the COVID-19 epidemic (hereinafter referred to as the “resolving method”). (3) The impact of the COVID-19 epidemic on students' life and study.

### 3.2. Establishment of BP Neural Network Model

**Figure 3:**
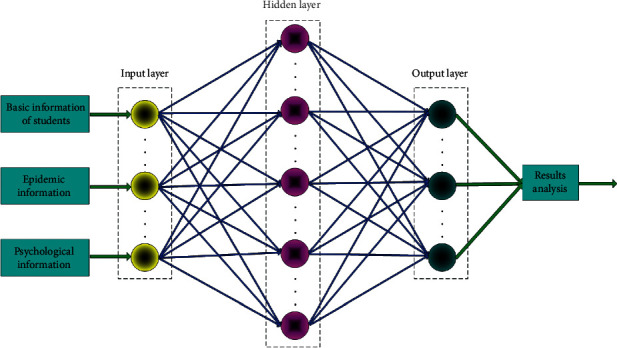
BP neural network model structure for analyzing the relationship between college students' psychological status and epidemic situation.

From the feasibility analysis, it is concluded that when BP neural network is applied to the research on the relationship between college students' psychological status and epidemic situation, it can effectively simplify the evaluation process and improve the evaluation efficiency and accuracy. In the process of creating the network model, it is necessary to maintain the good generalization ability of the model. In short, the network model must be able to widely adapt to different types of samples (i.e., different evaluation objects, indicators, etc.). Therefore, when constructing the BP neural network model, this paper will fully consider and reasonably set the key factors such as the structure, algorithm, number of neurons, and error accuracy of the network model (Figure 3).

In practical research, the number of neurons in the input layer depends on the number of variables contained in the problem. This study involves the evaluation index of the relationship between college students' psychological status and epidemic situation. According to the evaluation index system and the 10 secondary evaluation indexes involved in the questionnaire survey, this paper plans to record the number of neurons in the input layer as 10.

The output layer mainly depends on the actual needs of the studied object. The number of neurons in the output layer is selected after comprehensive analysis and judgment. In this study, the output content is the comprehensive evaluation result of the relationship between college students' psychological status and epidemic situation. Therefore, this paper sets the number of neurons in the output layer as 1.

The BP neural network with only one hidden layer is constructed this time. In order to ensure the effective training of the network model, the network structure needs to be simplified. Because the number of neurons in the hidden layer will directly affect the accuracy of network training, we need to be very careful when selecting the number of neurons. If the number of hidden layer neurons is too small, it will greatly reduce the fault tolerance of the network model and the accuracy of identifying samples. If the number of settings is too large, the network training time will be too long, and the fitting degree of the network model will be greatly increased, resulting in the problem of overfitting. We analyze or calculate the number of neurons contained in the input layer and output layer to determine how many neurons are needed in the hidden layer.

## 4. Experiment and Result

In this study, the convenience sampling method was used to investigate college students, and all respondents participated voluntarily. With the help of questionnaire star, the survey list is included in the electronic questionnaire. The questionnaire uses unified guidelines to introduce the purpose and significance of the survey. After checking the collected data, input it into SPSS 20.0 software for data analysis and processing. The count data is expressed in cases and percentages. *T*-test, analysis of variance, correlation analysis, and multiple linear hierarchical regression analysis were used to explore the related factors and their interaction.

The basic status of the undergraduates participating in the survey is shown in Tables 1 and 2. From the questionnaire on the basic information of students and questionnaire on basic information of students affected by epidemic situation, the survey samples in this paper meet the needs of statistical surveys, which shows that the results of the experimental survey conducted in this paper are statistically significant.

Table 3 shows the survey statistics of students' personal behavior during the peak period of the COVID-19 epidemic. It can be seen from the table that, at the peak of the COVID-19 epidemic, although there are quite a few survey subjects who have never attracted attention in terms of hygiene habits and improving autoimmunity, the proportion is lower than that of students who sometimes, often, and always maintain good hygiene habits and pay attention to improving their own immunity.

Shown in Table 4 is the survey statistics of the personal behavior of students after the COVID-19 epidemic. In the later period of the COVID-19 epidemic, 7.21% of the survey respondents occasionally washed their hands, 64% of the survey respondents occasionally, sometimes, or often spit and throw garbage, and 2.35% of the survey respondents often or always dine in small restaurants outside. 8.25% of the survey respondents have never had the habit of regular work and rest. Less than half of the survey respondents sometimes, often, or always exercise their bodies, and 44.87% of the survey respondents never or only occasionally pay attention to the combination of diet and nutrition. Only 14.9% and 1.73% of the survey respondents always work and rest on time and exercise every day, respectively.

At the peak of the COVID-19 epidemic, among 780 students, 273 sometimes or often wear masks, accounting for 35.02%. 237 people have taken medicine to prevent the COVID-19 epidemic, accounting for 30.48%, and 160 people have taken healthcare products to increase their resistance, accounting for 20.50%. 66.75% of the survey respondents once felt suffocated in public, among which 220 people felt suffocated sometimes or often, as shown in Table 5.

The middle and late stages of the COVID-19 epidemic are between the peak of the COVID-19 epidemic and the latter part of the COVID-19 epidemic. As shown in Table 5, the psychological status during this period is measured using five anxiety and three depression items. 47.69%, 65.75%, 60.48%, 92.75%, and 80.76% of students have had obvious feelings of anxiety, premonitions of misfortune, fatigue, akathisia, and sleep disorders, respectively. 61.88%, 42.79%, and 82.48% of the survey respondents had significant depression, irritability, and feeling of emptiness, respectively, as shown in Table 6.

The results of single-factor logistic regression analysis are shown in Table 7. Among them, the eight factors of gender, age, academic performance, peak behavior score, opinions on school measures to prevent the COVID-19 epidemic, resolving ways to confide in others, doing nothing, and coping style scores are significant.

This paper uses reliability to analyze the feasibility and effectiveness of the questionnaire. Reliability refers to the consistency and reliability of the results measured by the test or scale tool. In order to ensure the reliability of the questionnaire, this paper uses the Cronbach coefficient analysis method to analyze the internal consistency of the questionnaire data. For Cronbach coefficient the calculation formula is as follows:

(8) $$\begin{array}{c} \delta =\frac{M}{M-1}\left(1-\frac{\sum f_{i}^{2}}{f^{2}}\right), \end{array}$$

where *M* denotes the total number of items in the scale, *f*_*i*_ is the variance of score in question *i*, and *f* is the variance of the total score of all items. According to the formula and based on the data obtained from the questionnaire, the coefficient of this scale is 0.85, which proves that the questionnaire is stable and has high internal consistency and trust level.

Fit the logistic avoidance model of multinomial ordinal classification to the factors in Table 7. Set the screening standard to 0.15 and reject to 0.05. The results are shown in Table 8. The younger you are, the more satisfied you are with the measures taken by the school, the higher your peak epidemic behavior score, the lower your choice of resolving ways to confide in others, and the lower your coping score, the more psychologically nervous you will be during the peak of the COVID-19 epidemic.

The regression results are shown in Table 9. Students who scored lower in anxiety in the middle and late stages of the COVID-19 epidemic were more satisfied with the measures taken by the school and chose self-resolving methods and felt more relaxed in the later stages of the COVID-19 epidemic.

According to the above experimental results, the following suggestions are put forward. First, multiple coordination is done to jointly protect students' mental health. COVID-19 epidemic should be published through key roles such as various media channels and counselors. On the one hand, it should reduce unnecessary fear and help students master the necessary preventive knowledge. On the other hand, we can strengthen the health education of college students' lifestyle through a variety of information platforms to promote the generation and maintenance of their healthy behavior. Second, strengthen the construction of teachers and improve the combat effectiveness of epidemic prevention psychological work. In case of public health emergencies, students' cognition of epidemic situation knowledge is easy to deviate and students' psychological emotion is easy to fluctuate. Mental health education is facing new challenges. Therefore, it is essential to improve their psychological competence under the epidemic situation. Through online peer counselors' daily work discussion, supervisors are invited to conduct professional supervision and actively learn relevant knowledge of psychological maintenance under the epidemic, to lay a solid foundation for mental health work under the epidemic. In addition, popularize psychological epidemic prevention knowledge through mental health knowledge publicity, psychological network support, and psychological cloud classroom and improve students' psychological quality and open students' psychological self-help mode [20].

## 5. Discussion

As an important group in our country, the mental health of college students has always been a hot issue of social concern. The COVID-19 epidemic has affected the learning style, lifestyle, and communication styles of college students and also affected the mental health of college students to varying degrees. China's epidemic prevention and control is still in a tense stage of external defense import and internal defense rebound. The study life and interpersonal communication of college students will still be in a state of restraint and depression in the short term, and such long-term restraint and depression can easily lead to the transformation of some college students' mental health problems from nothing to existence, from small to large. Therefore, under the background of the COVID-19 epidemic, doing a good job in the mental health education of college students, preventing the occurrence of college students' psychological problems, and improving the level of college students' mental health are an urgent task for educators.

This paper combines the COVID-19 epidemic situation to investigate the current psychological emotions of college students through a questionnaire survey. Through investigation and analysis, it can be known that various behaviors of college students under public health emergencies are the result of a combination of multiple factors. This paper studies multiple factors in the research and combines factor analysis to finally get the influence of each factor on the psychology of college students.

## 6. Conclusion

In order to explore the impact of COVID-19 on college students' mental health, this paper proposes a BP neural network based model for the relationship between college students' psychological status and epidemic situation by evaluating the psychological status of college students and exploring the related factors. The relationship model constructed in this paper is used to analyze the psychological factors affecting college students, and the principal component analysis method is used to explore the factors affecting college students' psychology. From the model prediction and result analysis, it is known that the factors affecting college students' psychological status show multilevel and multifaceted characteristics. During the epidemic period, the psychological status of college students is not only related to demographic characteristics, but also affected by epidemic related factors. At the same time, their own behavior also has a more significant impact on their psychological status. On the premise of ensuring no infection, we should pay full attention to the intervention of college students' health behavior during the epidemic. In addition, the influence of COVID-19 on college students' psychology should focus on improving occupation quality, improving their physical quality, humanistic quality, and moral quality, and improving the stability of colleges and universities in public health emergencies. By evaluating the psychological status of college students and exploring the relevant influencing factors, this paper can provide reference for carrying out mental health education and formulating effective intervention programs.

## Figures and Tables

**Table 1:** Questionnaire on the basic information of students.

Project	Gender		Average age	Is it an only child		Physical conditions	
	Male	Female		Yes	No	Health	Unhealthy
Number of samples	482	298	20.52	502	278	758	22

**Table 2:** Questionnaire on basic information of students affected by epidemic situation.

Project	Whether to serve as a student leader		Epidemic situation in hometown		
	Yes	No	Serious	General	No
Number of samples	130	650	82	226	472

**Table 3:** Statistical table of the survey of the personal behavior of students during the peak period of the COVID-19 epidemic.

Item		Hygiene habits			Improve immunity		
		Hand washing frequency	Spitting/throwing garbage	Dining out	Reasonable diet	Standard schedule	Exercise
Never	Number of people	0	556	486	137	49	105
	Proportion (%)	0	71.25	62.35	17.56	6.32	13.52
Occasionally	Number of people	51	145	177	200	88	229
	Proportion (%)	6.52	18.6	22.65	25.61	11.26	29.31
Sometimes	Number of people	184	60	88	184	177	207
	Proportion (%)	23.54	7.65	11.26	23.58	22.69	26.54
Often	Number of people	510	10	15	177	301	192
	Proportion (%)	65.43	1.25	1.96	22.64	38.64	24.58
Always	Number of people	35	10	14	83	165	47
	Proportion (%)	4.51	1.25	1.78	10.61	21.09	6.05

**Table 4:** Statistical table of the survey of students' personal behaviors after the COVID-19 epidemic.

Item		Hygiene habits			Improve immunity		
		Hand washing frequency	Spitting/throwing garbage	Dining out	Reasonable diet	Standard schedule	Exercise
Never	Number of people	0	543	467	144	64	143
	Proportion (%)	0	69.58	59.84	18.52	8.25	18.35
Occasionally	Number of people	56	140	155	206	106	236
	Proportion (%)	7.21	18.01	19.85	26.35	13.65	30.21
Sometimes	Number of people	191	54	81	190	198	190
	Proportion (%)	24.53	6.98	10.35	24.35	25.35	24.35
Often	Number of people	518	10	18	164	295	198
	Proportion (%)	66.43	1.3	2.35	21.05	37.85	25.36
Always	Number of people	14	32	59	76	116	13
	Proportion (%)	1.83	4.13	7.61	9.73	14.9	1.73

**Table 5:** Statistical table of the survey of the psychological status of students in the early stage of the COVID-19 epidemic.

Item	Never		Occasionally		Sometimes		Often	
	Number of people	Proportion (%)	Number of people	Proportion (%)	Number of people	Proportion (%)	Number of people	Proportion (%)
Wear mask	120	15.35	197	25.31	190	24.32	273	35.02
Take medicine	542	69.52	168	21.54	41	5.32	28	3.62
Take supplements	620	79.52	119	15.23	16	2.1	25	3.15
Psychological depression in the crowd	259	33.25	300	38.51	174	22.35	46	5.89

**Table 6:** Psychological status of depression in the middle and late stages of the COVID-19 epidemic.

Item		No or rarely		Sometimes		Often		Always	
		Number of people	Proportion (%)	Number of people	Proportion (%)	Number of people	Proportion (%)	Number of people	Proportion (%)
Anxiety situation	Anxiety	408	52.31	285	36.52	62	7.98	25	3.19
	Unfortunate premonition	267	34.25	168	21.52	152	19.52	193	24.71
	Fatigue	308	39.52	336	43.12	103	13.25	32	4.11
	Can't sit still	57	7.25	212	27.12	337	43.25	175	22.38
	Sleep disorder	150	19.24	242	31.02	244	31.24	144	18.5
Depression situation	Depression	297	38.12	375	48.03	95	12.12	13	1.73
	Irritable	446	57.21	251	32.15	62	7.98	21	2.66
	Emptiness	137	17.52	297	38.14	242	31.02	104	13.32

**Table 7:** Results of single-factor logistic regression analysis of psychological conditions at the peak of the COVID-19 epidemic.

Influencing factors	Parameter value estimation	Standard error	*χ* ^2^	*P*	OR
Gender	0.20382	0.07828	6.85437	0.00929	1.23624
Age	−0.06555	0.02646	6.19342	0.01343	0.94637
Academic performance	0.08908	0.03868	5.35835	0.02151	1.10292
Peak behavior	0.06161	0.01172	27.98245	0.00002	1.07363
Opinions on the school	0.15736	0.04282	13.61965	0.00020	1.18069
Confide in others	0.21978	0.07777	8.07495	0.00475	1.25543
Doing nothing	−0.27937	0.14100	3.96880	0.04787	0.76558
Coping method	−0.10403	0.01667	39.21759	0.00004	0.91102

**Table 8:** Logistic multiple stepwise regression analysis results of psychological conditions at the peak of the COVID-19 epidemic.

Influencing factors	Parameter value estimation	Standard error	*χ* ^2^	*P*	OR
Intercept 1	−1.373196	0.653773	4.455918	0.036057	
Intercept 2	0.392284	0.650642	0.367135	0.552066	
Intercept 3	2.131201	0.652359	10.78074	0.001111	
Age	−0.071104	0.026664	7.182918	0.007777	0.94132
Opinions on the school	0.099384	0.044036	5.159282	0.024038	1.11403
Behavior at peak	0.086456	0.012524	48.512118	0.00020	1.09989
Confide in others	0.266539	0.079184	11.443603	0.000808	1.31502
Coping method	−0.1313	0.017271	58.107522	0.00004	0.88678

**Table 9:** Logistic multiple stepwise regression analysis results of psychological conditions in the late stage of the COVID-19 epidemic.

Influencing factors	Parameter value estimation	Standard error	*χ* ^2^	*P*	OR
Intercept 1	−1.29805	0.34017	14.70409	0.00010	
Intercept 2	−0.08797	0.33118	0.07121	0.79851	
Intercept 3	1.17433	0.33108	12.70964	0.00040	
Intercept 4	3.74811	0.33997	122.76671	0.00004	
Student leaders	−0.24684	0.08524	8.47723	0.00384	0.79083
The relationship between teachers	−0.15039	0.05232	8.37068	0.00414	0.86860
Anxiety	0.04020	0.01424	8.04233	0.00485	1.05141
Opinions on the school	−0.37905	0.04464	72.79363	0.00002	0.69387
Self-resolving	−0.29139	0.08141	13.00769	0.00030	0.75649

## Data Availability

The labeled datasets used to support the findings of this study are available from the corresponding author upon request.
